# Collision of herbal medicine and nanotechnology: a bibliometric analysis of herbal nanoparticles from 2004 to 2023

**DOI:** 10.1186/s12951-024-02426-3

**Published:** 2024-04-01

**Authors:** Sinan Ai, Yake Li, Huijuan Zheng, Meiling Zhang, Jiayin Tao, Weijing Liu, Liang Peng, Zhen Wang, Yaoxian Wang

**Affiliations:** 1 China-Japan Friendship Hospital, Beijing, China; 2 Beijing Key Laboratory for Immune-Mediated Inflammatory Diseases, Institute of Medical Science, China-Japan Friendship Hospital, Beijing, China; 3 Beijing Hospital of Traditional Chinese Medicine, Capital Medical University, Beijing, China; 4 Dongzhimen Hospital, Beijing University of Chinese Medicine, Beijing, China; 5 Beijing Friendship Hospital, Capital Medical University, Beijing, China; 6 Henan University of Chinese Medicine, Zhengzhou, Henan China

**Keywords:** Herbal nanoparticles, Herbal medicine, Traditional Chinese medicine, Nanoparticles, Bibliometric analysis, VOSviewer, Citespace

## Abstract

**Background:**

Herbal nanoparticles are made from natural herbs/medicinal plants, their extracts, or a combination with other nanoparticle carriers. Compared to traditional herbs, herbal nanoparticles lead to improved bioavailability, enhanced stability, and reduced toxicity. Previous research indicates that herbal medicine nanomaterials are rapidly advancing and making significant progress; however, bibliometric analysis and knowledge mapping for herbal nanoparticles are currently lacking. We performed a bibliometric analysis by retrieving publications related to herbal nanoparticles from the Web of Science Core Collection (WoSCC) database spanning from 2004 to 2023. Data processing was performed using the R package Bibliometrix, VOSviewers, and CiteSpace.

**Results:**

In total, 1876 articles related to herbal nanoparticles were identified, originating from various countries, with China being the primary contributing country. The number of publications in this field increases annually. Beijing University of Chinese Medicine, Shanghai University of Traditional Chinese Medicine, and Saveetha University in India are prominent research institutions in this domain. The Journal “International Journal of Nanomedicine” has the highest number of publications. The number of authors of these publications reached 8234, with Yan Zhao, Yue Zhang, and Huihua Qu being the most prolific authors and Yan Zhao being the most frequently cited author. “Traditional Chinese medicine,” “drug delivery,” and “green synthesis” are the main research focal points. Themes such as “green synthesis,” “curcumin,” “wound healing,” “drug delivery,” and “carbon dots” may represent emerging research areas.

**Conclusions:**

Our study findings assist in identifying the latest research frontiers and hot topics, providing valuable references for scholars investigating the role of nanotechnology in herbal medicine.

**Graphical Abstract:**

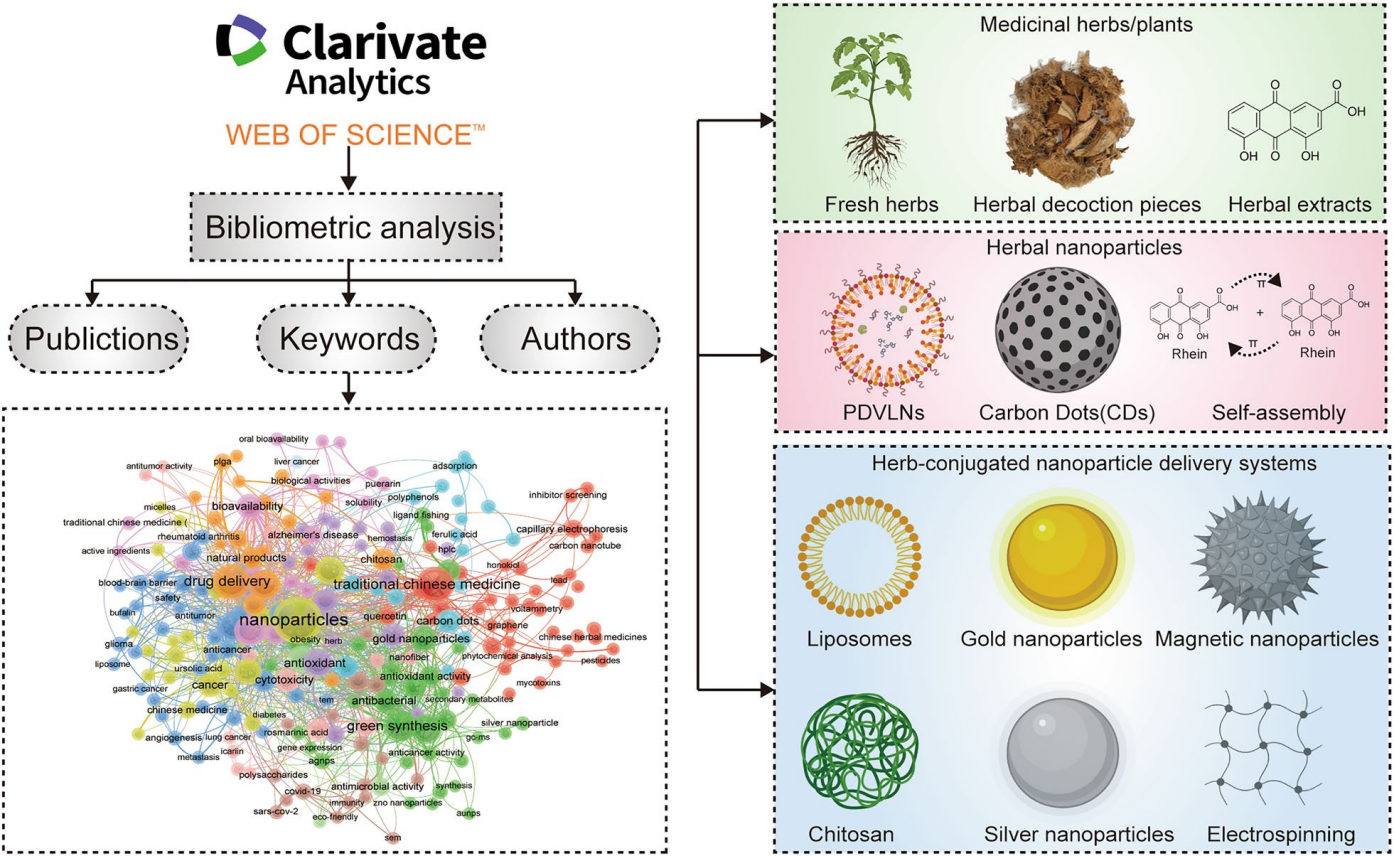

**Supplementary Information:**

The online version contains supplementary material available at 10.1186/s12951-024-02426-3.

## Background

Nanomaterials refer to solid, liquid, or gaseous materials with nanoscale dimensions, typically 1–100 nm. They possess unique physical and chemical properties, including high surface area, quantum effects, and size effects. Nanomaterials have wide applicability in various fields, including electronics, materials science, and biology [1]. Particularly noteworthy is their significant potential in the field of medicine, offering novel approaches and methods for disease diagnosis and treatment [2–6]. For example, the high affinity of nanomaterials for specific biomolecules enables cancer biomarker detection [7]. Furthermore, nanomaterials can optimize the treatment methods, including photodynamic therapy (PDT) and photothermal therapy (PTT), which are promising cancer treatment methods involving light-induced cell death. Combining nanotechnology with PDT and PTT can enhance treatment efficacy, using laser-induced heating of gold nanoparticles for targeted cell death induction or utilizing photosensitizers in conjunction with graphene oxide nanoparticles for a more effective PDT [8]. One of the extensively researched areas is the nanodrug preparation, wherein nanomaterial properties are leveraged to create drug delivery systems with high drug payloads, low toxicity, and controllable release. These nanoparticles not only possess therapeutic properties of their own but also serve as carriers that can transport drugs by altering their surface properties and size to control the rate and mode of drug release [9].

Herbs, known as medicinal plants, constitute a vital component of traditional medicine with a long history of therapeutic use. Traditional herbal medicines continue to be highly regarded and used in modern times in China, Japan, South Korea, India and Arab countries; traditional Chinese medicine (TCM) in China, Japan and South Korea, Ayurveda in India, and aromatherapy in Arab countries are prominent examples [10]. In particular, traditional Chinese herbs, holds an important role in complementary therapies in China as their precise efficacy and low toxicity are widely acknowledged. Over a hundred TCM institutions use herbal medicine for treating patients. The therapeutic potential of traditional Chinese herbal medicine has gained recognition by the World Health Organization, which has initiated a program aimed at promoting and researching the safety, efficacy, and quality standards of Chinese herbal medicine, enabling more countries to completely harness the therapeutic potential of traditional Chinese herbal medicine [11]. An increasing body of scientific research confirms the various biological effects of herbal medicine, including antitumor, anti-inflammatory, and antimicrobial properties [12–14]. Furthermore, herbal extracts provide extensive avenues for developing new medications, with many plant-derived drugs already in use [15], including artemisinin, a compound extracted from sweet wormwood that is widely used to treat malaria, berberine to treat diarrhea, and paclitaxel as a cancer chemotherapy drug [16–18]. The chemical components of medicinal plants and their wide-ranging applications will give rise to the development of innovative products with fewer side effects than the existing medications. However, herbal medicine has several challenges such as limited administration routes, poor water solubility, low bioavailability, inadequate targeting, and high toxicity that prevent several drugs from entering clinical use. Recently, nanotechnology has been widely applied to address these therapeutic challenges because of its advantages that include high penetrability, strong targeting capabilities, and efficient controlled release. The two key applications of nanotechnology in the field of traditional herbal medicine are nanosizing and nanodelivery systems. For instance, drug nanosizing can enhance drug solubility and stability, thereby improving the drug’s therapeutic effectiveness and reducing toxicity [19]. Furthermore, herbal nano-delivery systems enable precise drug delivery and controlled release, enhancing the drug’s efficacy and minimizing side effects through synergistic interactions with carriers [20]. The amalgamation of nanotechnology with traditional herbal medicine is the outcome of the intersection of traditional medicine and modern technology. Traditional medicine boasts a rich history, and nanotechnology provides new vitality into traditional herbs. To date, we have achieved a series of breakthroughs and advancements in the field of herbal nanoparticle research. The present study aims to summarize the research hotspots and progress in the field of herbal nanoparticles over the past 20 years, gain a better understanding of the role of nanotechnology in TCM, contribute to the modernization of TCM, and promote nanomedicine development in clinical applications.

Over the 20 years, substantial evidence has confirmed the critical role of nanotechnology in research and development of traditional herbs; several recent review articles have delved into various aspects of this theory [21–27]. However, a comprehensive analysis regarding the current state and future directions is currently lacking. Moreover, there is no objective description to highlight the research focal points in this field. Bibliometrics, introduced by Alan Pritchard in 1969, is a statistical method for analyzing literature information and identifying developmental trends as well as research hotspots in specific fields [28]. This method has been widely utilized in various disciplines, including medical research [29–33]. A bibliometric analysis of publications, countries, institutions, journals, authors, and keywords would enable a comprehensive understanding of the developmental process in the research field. Furthermore, for determining the foundations, frontiers, and highlights of this field, author keywords would be used to perform keyword co-occurrence analysis. To the best of our knowledge, bibliometric studies on the association between nanomedicine and coronary heart disease as well as breast, gastric, and colon cancers have already been conducted [34–38]. Although bibliometrics has not been applied to the study of nanotechnology and traditional herbs yet, the role of nanotechnology in traditional herbal medicine is indeed a shining gem in current research. Therefore, to fill this knowledge gap, the present study aimed to objectively reveal the current status and future directions of nanotechnology in traditional herbs via a bibliometric analysis; identify major contributors, institutions, countries, and current research focal points; review current research hotspots; and anticipate research trends and future prospects in this field.

## Methods

### Data source and search strategy

**Fig. 1:**
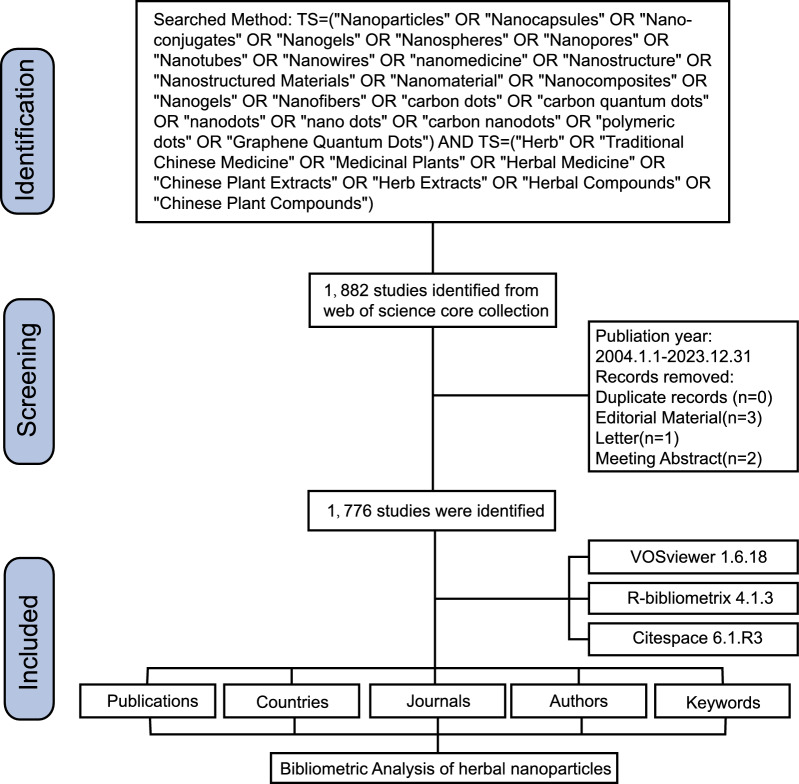
Flowchart of the literature screening process

We conducted an extensive search and analysis using the Web of Science Core Collection (WoSCC) database, covering English-language literature from 2004 to 2023 (up to December 31, 2023). Bibliometric analysis is generally performed using the WoSCC and Scopus databases; we selected WoSCC because its covers several renowned and influential journals, which are favored by academic researchers [37, 39]. We employed the following search term combinations: [TS = (“Nanoparticles” OR “Nanocapsules” OR “Nanoconjugates” OR “Nanogels” OR “Nanospheres” OR “Nanotubes, Carbon” OR “Nanopores” OR “Nanotubes” OR “Nanowires” OR “nanomedicine” OR “Nanostructure” OR “Nanostructured Materials” OR “Nanomaterial” OR “Nanocomposites” OR “Nanogels” OR “Nanofibers” OR “carbon dots” OR “carbon quantum dots” OR “nanodots” OR “nano dots” OR “carbon nanodots” OR “polymeric dots” OR “Graphene Quantum Dots”) AND TS = (“Herb” OR “Traditional Chinese Medicine” OR “Medicinal Plants” OR “Herbal Medicine” OR “Chinese Plant Extracts” OR “Herb Extracts” OR “Herbal Compounds” OR “Plant Compounds” OR “Phytochemicals”)] to retrieve all relevant records. Articles using a search period from 2004 to 2023 were selected for this study; this was considered a sufficient timeframe to reflect the current research status in the field as there was little to no literature on herbal nanomaterial formulations before 2004. All relevant literature concerning herbal nanoparticles was exported in TXT format as “Full Records and References.” Subsequently, it was imported into the R package bibliometrix, VOSviewer, and CiteSpace for a comprehensive bibliometric analysis. The workflow diagram is illustrated in Fig. 1.

### Data analysis

We used the bibliometric tools such as Bibliometrix R package, VOSviewer, and CiteSpace to visualize the results of our bibliographic analysis. To enhance the presentation of our research outcomes, we carefully selected suitable software for examining various facets of our study. In this investigation, we utilized R (version 4.1.2), R-bibliometrix package (version 3.0.3, http://www.bibliometrix.org), CiteSpace (version 6.1. R2), and VOSviewer software (version 1.6.18) to visualize our selected literature [36, 37, 39]. By leveraging these tools, we performed an indepth analysis of publications, countries, institutions, journals, authors, and keywords. Using the R-bibliometrix package, we performed frequency statistics for authors, journals, institutions, and countries, which included publication and citation counts. For each category, the top 20 frequencies were displayed. Furthermore, we created a word cloud of the top 50 author keywords in R-bibliometrix, wherein the word size represented their frequency. For the remaining software parameters, the default settings were used. During the execution of R-bibliometrix, we used Biblioshiny that serves as a user-friendly web interface for R-bibliometrix and facilitates the visual display of data analysis and social network graphs [40]. We used the powerful R package ggplot2 (version 3.4.2) to enhance the visual representation of our research findings further. Moreover, we used VOSviewer software (version 1.6.16, https://www.VOSviewer.com), developed by Nees Jan van Eck and Ludo Waltman at Leiden University [41], to generate co-authorship, country, institution, and keyword co-occurrence networks, with node size indicating the frequency of appearance or citation and connecting line thickness reflecting collaboration strength. For the keyword co-occurrence network, we set the minimum frequency of appearance to 10 for authors, institutions, journals, and keywords. For countries, the minimum appearance frequency was set at 5, and for article citation counts, the minimum frequency was set to 100. Furthermore, we used CiteSpace, a widely used bibliometric analysis and visualization software developed by Professor Chaomei Chen [42] to facilitate keyword cluster analysis and keyword cluster timeline visualization. We pruned slice networks and merged them using Pathfinder and performed keyword K-means clustering analysis using a G-index value of k = 10.

## Results

### Number of publications and citation evolution

**Fig. 2:**
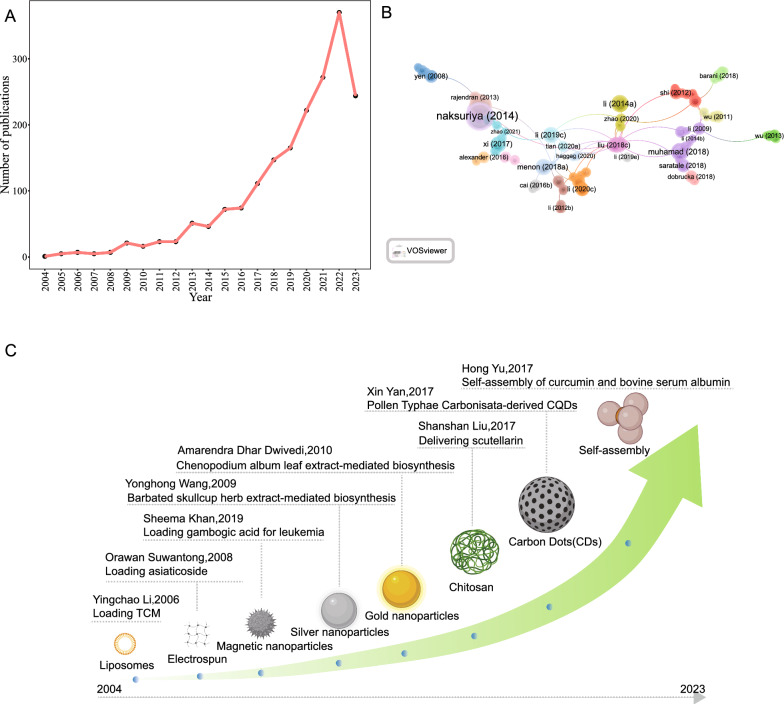
Analysis of all publications. **A** Trends in annual publications. **B** Co-citation network analysis of publications. **C** Chronological timeline of key articles in herbal nanoparticle research

In total, we included 1876 publications from the WoSCC database that comprised 1512 articles and 364 reviews. We depicted a line chart illustrating the evolution of the number of publications related to herbal nanoparticles from 2004 to 2023 (Fig. 2A). The entire period was divided into the following three phases based on the annual publication growth rate: first phase (2004–2008), marked a period of stability; second phase (2009–2016), experienced slow growth; and third phase (2017–2023), witnessed rapid growth.

According to VOSviewer’s co-citation network analysis (Fig. 2B), the most cited article in the WoSCC database was authored by Eileen White. This article, titled “Curcumin nanoformulations: a review of pharmaceutical properties and preclinical studies and clinical data related to cancer treatment” [43] was published in the *Biomaterials* journal (IF = 14.0) in 2014 and accumulated a total of 630 citations. The second most cited article was “Nanotechnology-based drug delivery systems and herbal medicines: a review” [20] by Bonifacio that was published in the *International Journal of Nanomedicine* (IF = 8.0) in 2014. By systematically assessing the citation counts of articles, we selected a series of pivotal papers that reported the applications of specific nanotechnologies in traditional herbal medicine at an early stage [44–51]. Subsequently, we constructed a chronological timeline, highlighting some milestone studies in a sequential manner (Fig. 2C).

### Core journals analysis

**Fig. 3:**
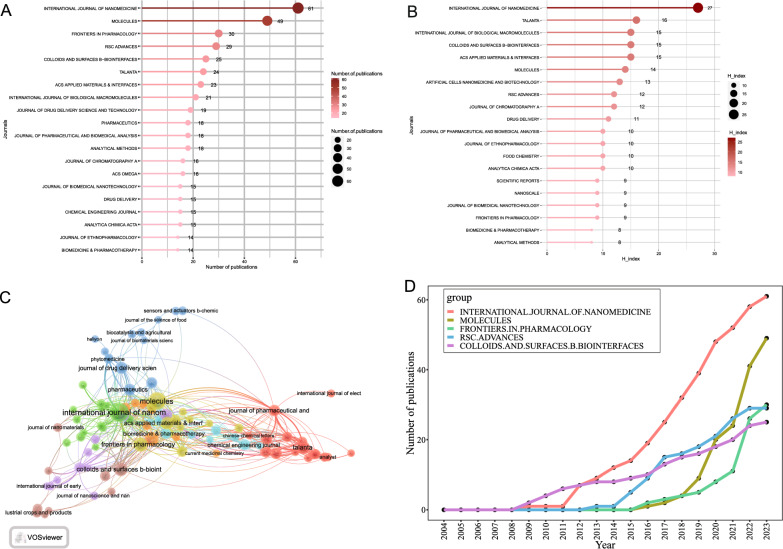
The most productive and influential journals in research on the herbal nanoparticles. **A** The top 20 most productive journals contributing to research. **B** The top 20 journals ranked by H-index. **C** The publication trends in the top 5 journals over time. **D** Co-citation network analysis of journals

Figure 3A shows the top 20 journals that published articles related to herbal nanoparticles from 2000 to 2022; these journals collectively published 1157 articles, accounting for 25.27% of the total publications. Among them, the *International Journal of Nanomedicine*, with an impact factor of 8.0 and a Journal Citation Reports Q1 ranking, published 61 relevant papers, contributing to 3.24% of all publications. The H-index is an indicator used to assess the journals’ quantity and impact [52, 53]. Figure 3B shows that the *International Journal of Nanomedicine* (H-index = 27) and *Talanta* (H-index = 16) are the two most influential journals based on their H-index. Furthermore, the journal co-citation network based on VOSviewer visualization (Fig. 3C) suggested that the three key journals with the highest total link strength were the *International Journal of Nanomedicine*, *Molecules*, and *Journal of Pharmaceutical and Biomedical Analysis*. Figure 3D shows that the growth trends of the five major journals indicate that the *International Journal of Nanomedicine* has consistently been a popular journal in this field.

### Analysis of countries and institutions

**Fig. 4:**
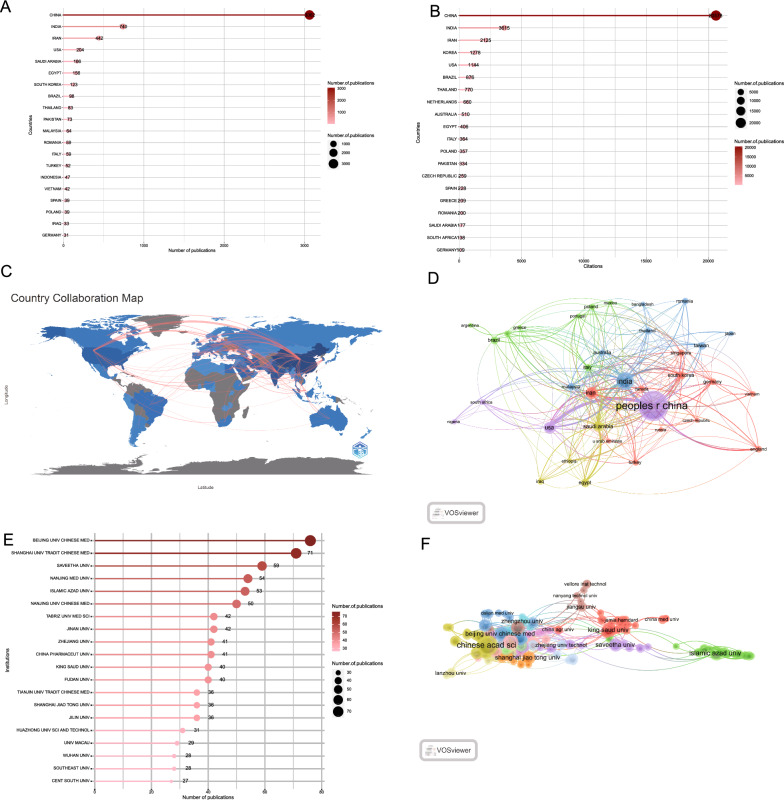
Contribution and collaboration among countries and institutions. **A** The top 20 most productive countries. **B** The top 20 most cited countries. **C** contribution and collaboration among countries. **D** Collaboration map with a minimum number of documents of a country of > 10 publications based on collaboration among countries. **E** The top 20 most productive institutions. **F** Collaboration map with a minimum number of documents of a country of > 5 publications based on collaboration among institutions

**Fig. 5:**
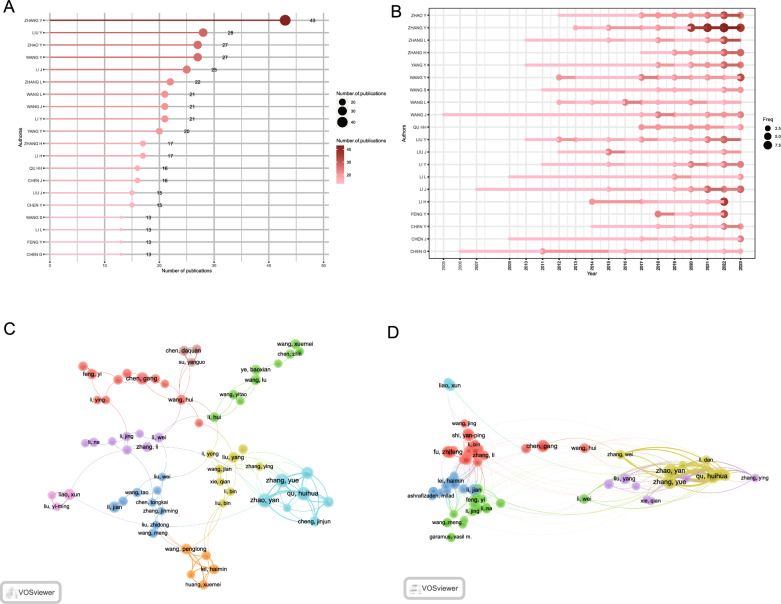
Authors’ contribution and collaboration. **A** The top 20 most productive authors. **B** The top 20 author’s production over time. **C** Authors’ collaboration network. **D** Co-citation network of authors

Figure 4A, B illustrate the number of articles published and the collaboration network among 87 countries conducting research on herbal nanoparticles. In terms of productivity, China leads with 3051 articles, followed by India (737 articles), Iran (442 articles), United States (203 articles), and Saudi Arabia (166 articles). Regarding their respective citation counts, China stands out as a prominent contributor in this research field, with a total citation count of 20,578, surpassing other countries such as India (10,506 citations), Iran (5296 citations), and South Korea (1278 citations). These countries have rich herbal usage traditions. China, especially, traces its herbal usage, which continues to the present day, back over 2000 years. Figure 5C, D, using VOSviewer and R-bibliometrix, illustrate the collaborative relationships among the leading countries, primarily demonstrating close collaborations and exchanges between China, India, and the United States. Among the 36 countries involved in international collaborations with a minimum of 5 articles, China has the highest number of collaborations with other countries (227), followed by India (167) and the United States (146).

These published articles originate from 2258 global institutions. Figure 5E lists the top 20 high-productivity institutions in herbal nanoparticle research, with 16 of them being located in China. The remaining four institutions include Saveetha University in India, Islamic Azad University in Iran, Tabriz University of Medical Sciences in the United States, and King Saud University in Saudi Arabia. The China Academy of Chinese Medical Sciences has the highest number of published articles (76) followed by Shanghai University of Traditional Chinese Medicine (71 articles), Saveetha University (59 articles), and Nanjing Medical University (54 articles). The collaboration network in Fig. 5F highlights the most influential institutions, including the Chinese Academy of Sciences (71 links), China Pharmaceutical University (39 links), and Fudan University (37 links).

### Author contributions

In total, 8234 authors contributed to the literature on herbal nanoparticles, with an average of 4.38 authors/article. The top 20 most prolific authors collectively contributed to 196 articles, accounting for 22.27% of all published work (Fig. 5A). Among these authors, Yan Zhao from Beijing University of Chinese Medicine stands out as the most prolific, contributing 19 articles in this field, followed closely by Yue Zhang (16 articles) and Huihua Qu (16 articles) from the same research team. Figure 5B illustrates the high productivity of the top 20 authors, indicating their regular publication patterns and demonstrating their strong commitment to this research area. To identify the key authors and study collaboration patterns, we used VOSviewer to create a collaboration network (Fig. 5C). For clarity, the collaboration network focuses on displaying collaborations among 116 authors who were involved in a minimum of 5 articles. The network illustrates close collaborations among the authors, each with a centrality of < 0.1. Within the collaboration network, Yan Zhao demonstrates the most significant collaborative relationships, with a link strength of 67. Subsequently, Huihua Qu (65) and Yue Zhang (65) also emerge as notable collaborators in the network. A co-citation network analysis described the collaborative relationships among 80 authors involved in at least 5 articles (Fig. 5D). The co-citation analysis further underscores Yan Zhao, Huihua Qu, and Yue Zhang as the top three authors in this context.

### Keyword and hotspot analysis

**Fig. 6:**
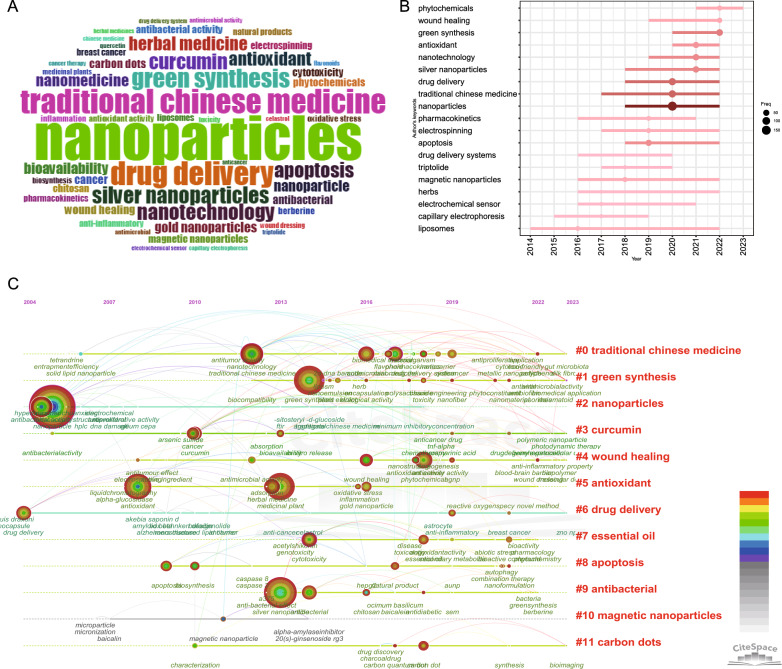
Analysis of topic trends. **A** Word cloud based on author’s keyword. **B** The keyword cluster timeline. **C** Trend topics based on author’s keywords over time

**Fig. 7:**
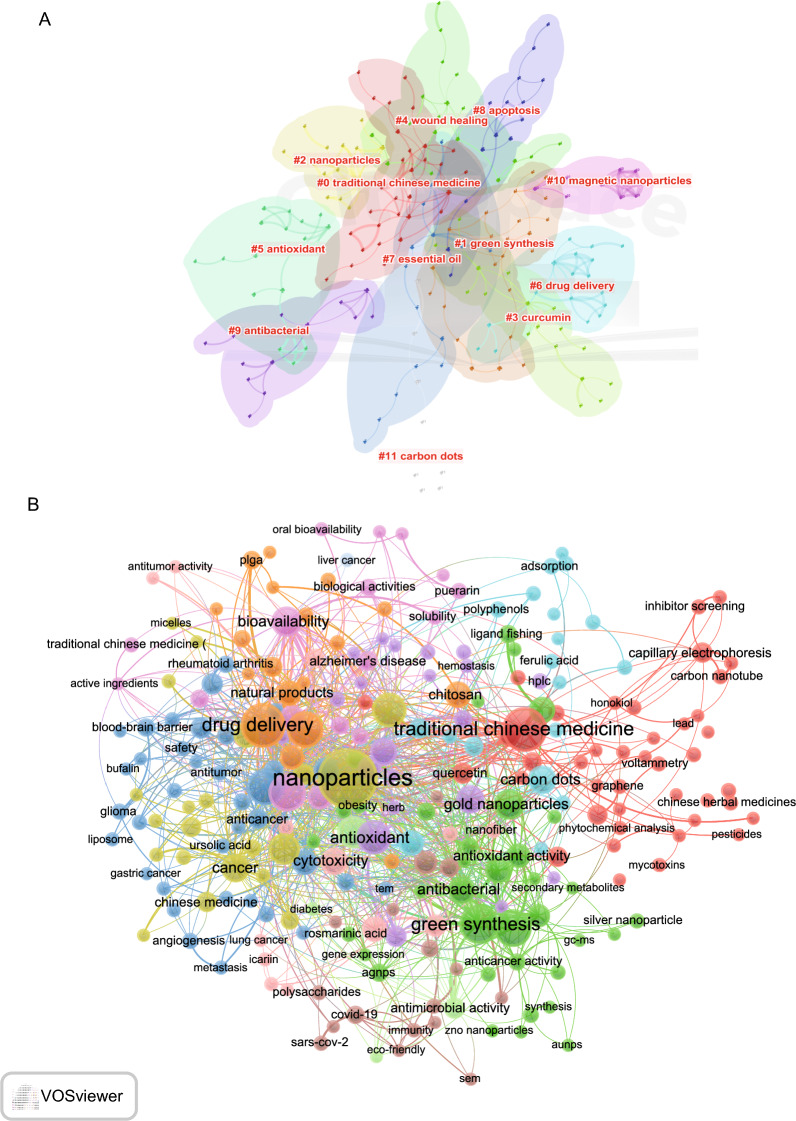
Keywords cluster analysis. **A** Keywords cluster analysis conducted using CiteSpace. **B** Co-occurrence visualization network of keywords

A comprehensive keyword analysis on the selected 1876 articles related to herbal nanoparticles was performed using “Author Keywords” from the Biblioshiny application and “Keywords Plus” provided by the Thomson Reuters editorial team. In total, 5134 keywords were identified. However, upon comparing the results from these two sources, “Author Keywords” was observed to provide more accurate results, making it the primary data source for the analysis. The frequency of each keyword was visually represented using a word cloud, as shown in Fig. 6A. The term “nanoparticles” had the highest frequency, mentioned 151 times, followed by “traditional Chinese medicine” (82 times) and “drug delivery” (80 times). Notably, high-frequency keywords that have emerged in recent years are displayed in Fig. 6B. Figure 6C shows the keyword cluster timeline visualization, highlighting the recent research hotspots, including TCM, green synthesis, nanoparticles, curcumin, wound healing, drug delivery, and carbon dots (CDs). We conducted a keyword analysis on the selected 1876 articles using VOSviewer and CiteSpace software. To examine the association among keywords and identify prevalent topics, we performed a co-occurrence analysis. Using CiteSpace, we categorized these keyword clusters into 11 categories, including TCM, green synthesis, nanoparticles, curcumin, wound healing, antioxidants, drug delivery, essential oil, apoptosis, antibacterial, magnetic nanoparticles, and CDs (Fig. 7A). Similarly, using VOSviewer, we selected the top 205 keywords that appeared at least 10 times, constructed a co-occurrence network (Fig. 7B), and identified clusters represented by different colors, including red, green, blue, and yellow. The nodes of the same color within a cluster represent closely related co-occurrences, with node size and link width varying based on the degree and strength of co-occurrence. “Betweenness centrality” is used to measure the importance of nodes in the network, with larger nodes and higher total link strength indicating more informative contributions. The two largest nodes with the highest total link strength were “nanoparticles” and “traditional Chinese medicine” within the yellow cluster. The top 20 keywords include nanoparticles, TCM, drug delivery, green synthesis, silver nanoparticles, nanotechnology, curcumin, apoptosis, antioxidants, herbal medicine, nanomedicine, nanoparticles, bioavailability, gold nanoparticles, cancer, CDs, wound healing, antimicrobial, and plant compounds. Additional file 2: Table S1 provides specific information for each cluster of keywords.

## Discussion

Recently, with the advancement of science and technology, the importance of nanomaterials in the field of medicine has garnered increasing interest, positioning it as a significant focal point in research. Furthermore, traditional herbal medicine, with the assistance of nanotechnology, has piqued the curiosity of more researchers. This is the first study that has attempted to use bibliometric analysis methods for investigating publications related to herbal nanoparticles. We utilized R software, VOSviewer, and CiteSpace to conduct a bibliometric visual analysis of 1876 herbal nanoparticle related documents extracted from the WoSCC database. The results of this study provide a comprehensive understanding of the research trends in herbal nanoparticles from 2004 to 2023. Based on publication output trends, since 2010, the number of articles related to herbal nanoparticles has steadily increased, with explosive growth occurring since 2016. This may be attributed to the publication of key articles that have driven interest in this field. An analysis of journal publications revealed considerable enthusiasm among international journals, especially in specialized journals such as the *International Journal of Nanomedicine*, for exploring the functionality of herbal nanoparticles. This indirectly reflects the strong interest of prestigious journals in this research direction. China has emerged as a major research powerhouse in this field, leading in research output [54, 55]. This can be attributed to China’s rich traditional use of herbal medicine that dates back centuries [56]. Moreover, the Chinese government has been increasing its financial support for studies related to herbal nanoparticles. Chinese researchers have published a substantial volume of research papers on herbal nanoparticles in domestic and renowned international journals, highlighting the vibrant development of this field [21]. Consequently, China’s research output and influence in this area have been increasing. Furthermore, India, as one of the earliest nations to use herbal medicine and with its traditional Ayurvedic medicine system centered around herbal usage [57]. Similarly, we can observe the utilization of plant extracts for medical purposes in regions such as the Arab countries because of their use in aromatherapy [58]. China’s neighboring countries, such as Japan and South Korea, engage in cultural exchanges related to TCM, contributing to their leading positions in research on herbal nanoparticles [59, 60]. Furthermore, extensive collaborations have been established between China, India, and the United States. Although the United States has published fewer articles as compared to China and India, it engages in more collaborations between these two countries. The advanced facilities in the United States promote research and development in the field of herbal nanoparticles via these collaborations. Currently, international co-operation in this field remains in its nascent stage and should be expanded further to promote the global dissemination of herbal nanoparticle research.

**Fig. 8:**
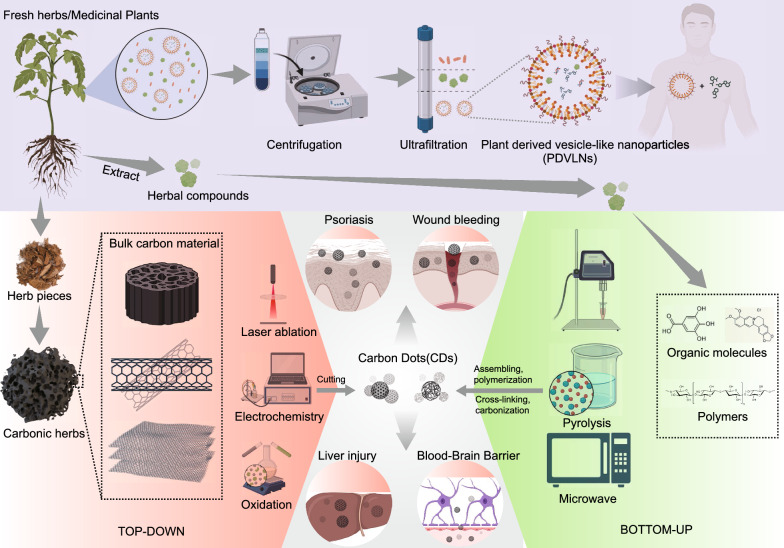
Natural herbal nanoparticles and carbon dots

**Fig. 9:**
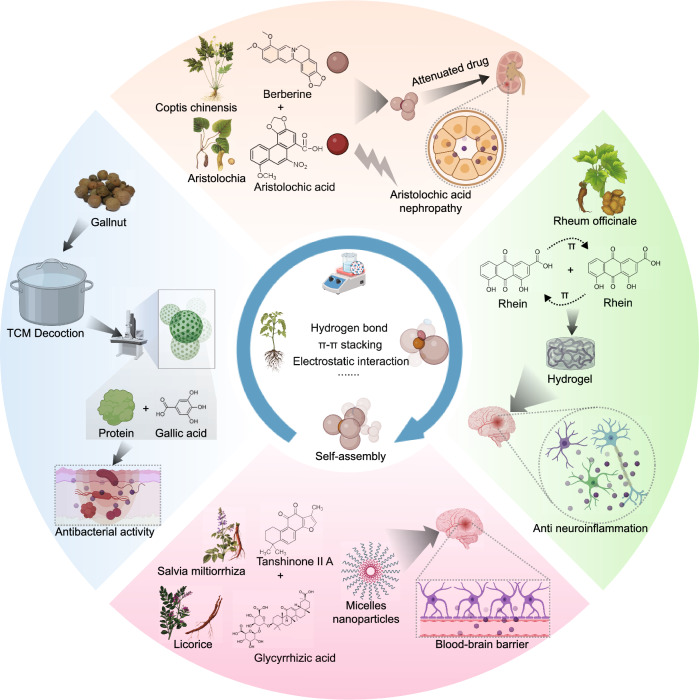
Self-assembled herbal nanoparticles

**Fig. 10:**
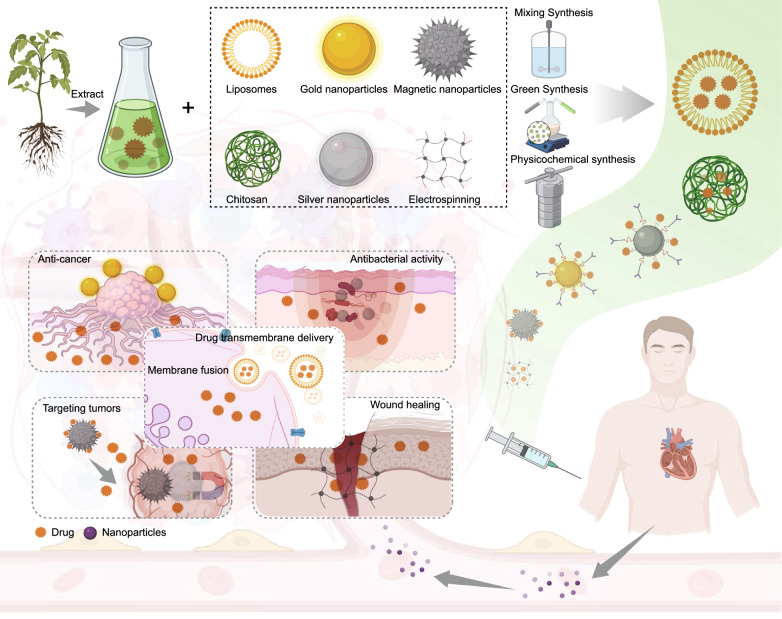
Herbal nanoparticle delivery systems

Based on the results of the author’s analysis, Yan Zhao emerges as the most prolific researcher who actively collaborates with several other researchers. Yue Zhang and Huihua Qu, who rank second and third, respectively, belong to the light blue region of the author collaboration network and are part of the same team at Beijing University of Chinese Medicine. They have conducted extensive research on the biological activities of CDs from TCM. Their work has uncovered applications of these CDs in various biological functions, including hemostasis, anti-inflammatory properties, and hepatoprotection [61–64]. Researchers in the yellow region, including Penglong Wang, Haimin Lei, and Xuemei Huang, have focused on the self-assembly structures and supramolecular nanostructures of natural medicines [65, 66]. In the brown region, Professor Daquan Chen and his team from Yantai University primarily conducted studies on targeted nanodelivery systems for TCM and their extracts [67–69]. They explored various systems, including plant-derived exosome-like vesicles, macrophage membrane biomimetic nanoparticles, and nanomicelles, for delivering natural bioactive products. In the red portion, Professor Gang Chen from Guangdong Pharmaceutical University investigated the green synthesis of silver nanoparticles using TCM and achieved nanodelivery of TCM [70]. Based on the current keyword analysis, the research hotspots in the field of herbal nanoparticles primarily encompass several aspects of nanoparticle synthesis methods, types, and herbal compounds. We categorized the keyword hotspots into the following five aspects: natural herbal nanoparticles, CDs, self-assembled herbal nanoparticles, herbal nanoparticle delivery systems, and herbal compounds. We reviewed the research focus areas for each category. Figures 8 and 9 summarize natural herbal nanoparticles, CDs, and self-assembled herbal nanoparticles. Figure 10 provides a comprehensive overview of the focal points of herbal nanoparticle delivery systems. Finally, Table 1 presents research information on the top 10 herbal compounds related to nanoparticles [71–149]. We hope our data will provide valuable insights into the most promising hotspots in herbal nanoparticles.

**Table 1:** Research information of top 10 herbal small molecule compounds associated with nanoparticles

Herbal compounds	Structural formula	Molecular formula	Molecular weight	Solubility	Nanoformulation	Biological activity	References
Curcumin		C_21_H_20_O_6_	368.38	Insoluble in water	Natural turmeric-derived nanovesicles	Colitis	[65, 66]
					Chitosan-based nanoparticle	anti-lung cancer	[67]
					Magnetic nanoparticles	Osteoarthritis; Acute Lung Injury	[68, 69]
					Cu2-xSe-based nanoparticles	Parkinson’s Disease	[70]
					Copper-curcumin nanoparticles	Rheumatoid Arthritis	[71]
					Self-assembly nanoparticles	Antioxidant activity	[72]
					silver nanoparticles	Anti-bacteria	[73]
Berberine		C_20_H_18_NO_4_^+^	336.36	Insoluble in water	Liquid Crystalline Nanoparticle	Non-Small-Cell Lung Cancer	[74]
					Natural Coptidis Rhizoma-derived nanovesicles	Improving the pharmacokinetics	[75]
					Liposomes	Photodynamic immunotherapy resistance	[76]
					Macrophage-derived exosomes	Spinal cord injury	[77]
					Polymeric nanoparticles	Cardio-metabolic diseases	[78]
					Chitosan	Ulcerative Colitis	[79]
					Polymeric Micelles	Rheumatoid Arthritis	[80]
					Self-Assembly of gallic acid and berberine	Angiogenesis; Anti-inflammatory; Antibacterial	[81]
					Self-Assembly of berberine and hesperetin	Ulcerative Colitis	[82]
					Self-assembled of berberine derivatives and rhamnolipids	Anti-Helicobacter pylori	[84]
					Self-Assemblies of berberine and Cinnamic Acid	Antibacterial	[85]
Celastrol		C_29_H_38_O_4_	450.61	Insoluble in water	Polymeric nanoparticles	Corneal Stromal Fibrosis; Antiobesity; Corneal allograft	[86, 87, 93]
					Inflammation-targeted polymeric nanomicelles	Rheumatoid arthritis	[88]
					Mesangial cells-targeted albumin nanoparticle	Mesangioproliferative glomerulonephritis	[89]
					Enzyme-responsive nanoparticles	Arthritic joints	[90]
					ROS-responsive polymeric nanoparticles	Rheumatoid arthritis	[91]
					Nanoemulsion	Melanoma	[92]
					Self-assembled polyethylene glycols	Anticancer	[94]
					Silk Fibroin Nanoparticles	Pancreatic Cancer	[95]
Quercetin		C_15_H_10_O_7_	302.24	Insoluble in water	Cu2- x Se-based nanoparticles	Parkinson’s Disease	[105]
					Magnetic nanoparticles modified exosome	Type 2 Diabetes Mellitus	[97]
					Octahedral ceria nanoparticles	Periodontal Inflammation	[98]
					A Glutathione-activated Self-assembled nanoparticles	Anticancer	[100]
					Chitosan	Mucositis; Antioxidant; Antibacterial	[99, 101]
					Self-assembly of quercetin with amino acids and metal ion	Alzheimer’s disease	[96]
					Self-assembled quercetin-Fe3 + nanoparticles	Anticancer	[102]
					Self-assembled quercetin supramolecular nanoribbons	Intestinal inflammatory diseases	[103]
					Assembling Quercetin-ferrum nanoparticles	Photothermal therapy	[104]
Triptolide		C_20_H_24_O_6_	360.40	Insoluble in water	A Glutathione-activated Self-assembled nanoparticles	Anticancer	[100]
					Inflammation-Targeted Polymeric Micelles	Collagen Induced Arthritis	[105]
					Engineered exosomes	Melanoma	[106, 107]
					Dendritic cell-derived exosomes	Colitis; rheumatoid arthritis	[108]
					Light-activatable liposomes	Anti-hepatocellular carcinoma	[109]
					polymeric nanoparticles	Collagen-induced arthritis	[110]
					Hydrogels	Rheumatoid Arthritis	[111]
					Metal–organic framework	Melanoma	[112]
Baicalin		C_21_H_18_O_11_	446.36	Slightly soluble in water	Assembly of Baicalin	Anti-Obesity	[113]
					Self-Assembly of baicalin and sanguinarine	Antibacterial and wound healing	[83]
					Macrophage-targeting polymeric nanoparticles	Melanoma	[114]
					Polymeric nanofiber	Vascularized bone regeneration	[115]
					Hydrogel	Calvarial Bone Repair	[116]
					Carbon dots	Anticancer	[117]
Emodin		C_15_H_10_O_5_	270.24	Insoluble in water	Adipocytes-targeting polymeric nanoparticles	Anti-Obesity	[118]
					Ferrimagnetic polymeric micelles	Magnetic hyperthermia-chemotherapy	[119]
					Biomimetic nanocrystals	Anticancer	[120]
					Self assembly of emodin and albumin	Photodynamic Therapy	[121]
					Self-assembling Nanofibers	Retinal Ischemia–Reperfusion Injury	[122]
Puerarin		C_21_H_20_O_9_	416.38	Slightly soluble in water	Chitosan	Antimicrobial peptides; Wound healing in diabetes	[123, 124]
					Hydrogel	Uveal Melanoma; Heart Repair	[125, 126]
					Selenium-layered nanoparticles	Antidiabetic	[127]
					Tetrahedral Framework	Osteonecrosis	[128]
Resveratrol		C_14_H_12_O_3_	228.24	Insoluble in water	Biomimetic nano-delivery system	Colorectal cancer	[129]
					Polymeric nanoparticles	Alleviates Alzheimer's Disease; Neuroprotective effect; Macular degeneration	[130, 131, 134]
					Nanoparticles prepared by polysaccharides	Antioxidant;Anticancer	[132]
					tetrahedral framework nucleic acids	Diabetic Peripheral Neuropathy	[133]
					Macrophage exosomes	Multiple sclerosis	[135]
					Chitosan	Antibacterial; Antioxidant activity; Cholesterol-lowering	[136, 137]
					Polymeric nanoparticles	Rheumatoid arthritis	[138]
Rosmarinic acid		C_18_H_16_O_8_	360.31	Soluble in water	Natural Rosmarinic Acid-derived nanovesicles	Inflammatory Bowel Disease	[139]
					Self assembly of rosmarinic Acid	Synergistic Photoimmunotherapy	[140]
					Polymeric nanoparticles	Inflammatory bowel disease	[141]
					Electrospinning nanofibers	Wound healing	[142]
					Chitosan	Antioxidant; Anti-inflammatory; Photoprotective properties	[143]

### Natural herbal nanoparticles

Plant-derived vesicle-like nanoparticles (PDVLNs) are natural nanoparticles produced by plants [150]. Similar to the structure of animal exosomes, certain vesicle-like structures secreted by plants are nanoscale biological particles encapsulated within a phospholipid bilayer. They contain lipids, proteins, nucleic acids, and small molecules. These components have the ability to be transferred to recipient cells, thereby regulating intercellular communication. Even before research on mammalian-derived exosomes, plants could generate extracellular vesicles. However, progress in research on this topic has been slow. Early research predominantly focused on the functions of extracellular vesicles within plants, overlooking the potential for these nanoscale vesicles to transfer various bioactive substances among different organisms. In a previous review article, Zheng Lei proposed defining vesicle-like nanostructures isolated from plants as PDVLNs. PDVLNs have garnered considerable attention owing to their exceptional bioavailability, stability, and bioactivity. They hold promising therapeutic value and have become a central focus of interdisciplinary scientific research. The methods for preparing PDVLNs primarily include tissue disruption and tissue infiltration centrifugation methods. Further purification of PDVLNs can be achieved via density gradient ultracentrifugation, ultrafiltration, immunoprecipitation, and other techniques [150]. For example, previous research has demonstrated that garlic chive-derived vesicle-like nanoparticles extracted from garlic and Chinese chives, along with their active component phospholipid 1, 2-dilinoleoyl-sn-glycero-3-phosphocholine, exhibit the capability to inhibit NOD-like receptor thermal protein domain associated protein 3 inflammasome activation, thereby improving the metabolic health of diet-induced obese mice [151]. Modern medicine has confirmed that lemon water can effectively intervene in the occurrence and development of kidney stones. Zhang et al. successfully extracted lemon-derived extracellular vesicles (LEVNs) via density gradient centrifugation. They confirmed that LEVNs are delivered intact to the kidneys by natural oral administration. LEVNs restore calcium homeostasis and alter calcium oxalate crystal formation, thus helping mitigate stone aggregation and formation [151]. Another previous study extracted and purified exosomes from *Taraxacum officinale* through ultracentrifugation. *T. officinale*-derived exosomes alleviated intestinal tissue inflammation and minimized intestinal tissue damage, thereby significantly reducing high blood pressure in a rat model [152]. Although research on PDVLNs is currently limited, they hold the potential to become a major focus of future investigations.

### Carbon dots

CDs are carbon-based nanoparticles with sizes < 10 nm, featuring a variety of functional groups on their surfaces. They can be applied in various fields, including biomedicine and energy. In a comprehensive review published in *Nature Nanotechnology*, Luka Ðorđević and colleagues provide a systematic overview of how CD properties can be tailored for diverse applications via different chemical strategies and synthesis methods [153]. When TCM are subjected to high-temperature carbonization, they produce “traditional Chinese medicine carbonaceous nanoparticles” with particle sizes of < 10 nm, also known as “traditional Chinese medicine-derived carbon quantum dots.” These represent a novel class of carbon-based nanomaterials. These carbon quantum dots can be sourced from various TCM formulations, including Xueyu, Puhuang, and Baizhu charcoal, which typically have hemostasis, antidiarrheal, and astringent effects following their preparation. Currently, the preparation of TCM CDs primarily involves the following two approaches: “top–down” method, which uses physical or chemical means to isolate carbon materials; and “bottom–up” approach, which involves the polymerization or carbonization of small organic molecules [153]. Furthermore, TCM CDs retain the inherent pharmacological activities of medicinal materials while exhibiting biocompatibility, optical characteristics, and high stability. Weikang Luo and his team led the development of nontoxic, functional nanoCDs derived from inexpensive ingredients, such as peach kernels and safflower. These nanoCDs have been reported to penetrate the blood–brain barrier more easily in mice with traumatic brain injury, promoting neural function recovery, reducing brain edema, and minimizing neuronal damage [154]. Another study discovered that *Lycium barbarum* CDs can inhibit aging by modulating the m6A modification of Clip3, thereby alleviating radiation-induced bone damage [155]. A research team at the Beijing University of Chinese Medicine performed a series of investigations on TCM CDs using various medicinal plants, such as Puhuang charcoal, Longgu (fossilized bone), Gegen (Pueraria), Baishao (peony root), and Huangbapipi (Cortex Phellodendri), for a range of applications, including hemostasis, anxiety reduction, hyperuricemia management, hepatoprotection, and psoriasis treatment [61–64, 156]. In summary, TCM CDs represent a promising new class of nanomedicines with various potential applications.

### Self-assembled herbal nanoparticles

Self-assembly is a molecular-level phenomenon, wherein disordered molecular structures spontaneously arrange into ordered structures via noncovalent forces, including hydrogen bonds, van der Waals forces, and π–π, electrostatic, hydrophobic, and co-ordination interactions. In the process of decocting TCMs, self-assembly phenomena are widely observed, resulting in the formation of several nanoscale particles. For example, Han Bo reported the presence of spherical nanoparticles in gallnut decoction. These nanoparticles were formed via the self-assembly of gallic acid compounds and gallnut larval proteins [157]. Furthermore, TCM mixtures of Qingxue Chushi decoction contain drug delivery systems based on liquorice proteins. These systems can combine with compounds, including baicalin, paeoniflorin, and glycyrrhizic acid, to form nanoparticles, helping in the improving the skin conditions in a psoriasis mouse model [123]. Moreover, researchers have extracted extracellular vesicle nanoparticles from the TCM Jianpi Jiedu Recipe decoction. These nanoparticles were found to influence fibroblast activation, thereby inhibiting the growth and metastasis of colon cancer [158]. Furthermore, boiled liquorice proteins can self-assemble into nanoparticles, which have a potential for in vivo drug delivery for various medications. By encapsulating astragaloside IV in licorice protein nanoparticles, the solubility of astragaloside IV can be significantly improved [159]. The self-assembly phenomena in TCM decoction processes have driven researchers to develop a range of strategies for synthesizing nanoscale particles from the small molecules of TCM. For example, Professor Lei Haimin from Beijing University of Chinese Medicine has proposed a strategy for heteroline supramolecular self-assembly based on berberine. This strategy revealed that effective components of flavonoids in coptis detoxification decoction can self-assemble with berberine to form supramolecular structures. This alteration affects their inhibitory effects on *Staphylococcus aureus* and their ability to remove biofilms [66]. Furthermore, the self-assembly of aristolochic acid and berberine forms a linear heterogeneous supramolecule, which can reduce the acute nephrotoxicity of aristolochic acid, providing a new strategy for addressing toxicity issues in TCMs containing aristolochic acid [160]. Moreover, researchers have designed a biomimetic nanodelivery system that utilizes tanshinone IIA and glycyrrhetinic acid to self-assemble into nanomicelles, effectively accumulating drugs into the glioblastoma lesions [161]. Zheng had observed that rhein self-assembles into supramolecular hydrogels through intermolecular π–π interactions and hydrogen bonds. Compared to free drugs, these hydrogels more readily enter cells, subsequently achieving anti-neuroinflammatory therapy by inhibiting the Toll-like receptor 4/nuclear factor-κB signaling pathway [162]. In another study, salvianolic acid B and NapFFKYp could form a spontaneous hydrogel that promoted wound healing [163].

### Herbal nanoparticle delivery systems

Herbal nanoparticle delivery systems are a significant focus in the field of herbal nanoparticles. Keyword analysis indicates that the primary delivery carriers currently employed include gold nanoparticles, silver nanoparticles, magnetic nanoparticles, electrospinning, liposomes, and chitosan. Nanoencapsulation strategies involve enclosing the active ingredients of herbal medicines within nanoscale carriers to enhance their solubility, stability, and bioavailability, which in turn improves the therapeutic efficacy while reducing the potential side effects. Common encapsulation strategies include liposomes and chitosan. Liposomes, as a carrier system, have long been used in herbal nanodelivery. A previous study demonstrated that liposome encapsulation significantly enhanced berberine’s solubility in a buffer solution and helped maintain the ejection fraction following myocardial infarction. This suggests that employing liposome encapsulation significantly increases its therapeutic availability, indicating liposome encapsulation as a promising approach [164]. Recently, chitosan encapsulation has gained increasing attention in herbal nanodelivery research. Chitosan, a semisynthetic polysaccharide derived from chitin, has gained prominence as an ideal pharmaceutical carrier material because of its excellent biodegradability, biocompatibility, lack of immunogenicity, nonirritating nature, nonthermal properties, and nontoxicity. In this research trend, Nayeresadat Hasheminejad successfully prepared chitosan nanoparticles using an emulsion-ion gelation method to encapsulate clove essential oil. This strategy effectively enhanced the antimicrobial effects of clove essential oil, and in vitro release studies revealed precise drug release over 56 days [165]. In another study, Lazer et al. covalently bound folic acid to chitosan molecules, which resulted in the synthesis of chitosan–folic acid–quercetin nanoparticles. These nanoparticles exhibited anticancer effects on colon cancer by regulating the expression of pro-apoptotic genes, inhibiting tumor colony formation, and inducing cell apoptosis [166]. Apart from the therapeutic actions of herbal compounds, certain herbal ingredients served as targeted delivery agents. In this system, glycyrrhetinic acid-modified chitosan served as a carrier for the chemotherapy drug doxorubicin and was loaded into a metal–organic framework. The liver-targeting ligand glycyrrhetinic acid specifically bound to glycyrrhetinic acid receptors overexpressed on liver cancer cell membrane, providing an active targeting effect for the delivery system [167].

Furthermore, gold and silver nanoparticles have been used for herbal delivery, wherein drug molecules are loaded onto the surface of these nanoparticles via covalent and noncovalent interactions. However, their safety has been a subject of debate. Gold nanoparticles are widely used in cancer therapy, achieving multitarget treatment of tumors when combined with herbal medicines. In one study, curcumin and gold nanoparticles were used for treating cancer. These nanoparticles entered the body and bound to specific areas. By irradiating this region with lasers, gold nanoparticles were activated to generate heat, thereby destroying the cancer cells. Simultaneously, curcumin attached to the gold nanoparticles was released inside the body, seeking out other cancer treatment targets [168]. Silver surfaces possess antibacterial properties, and silver nanoparticles have significantly increased antibacterial efficacy because of their enlarged surface area. In this research, aqueous Siberian ginseng extracts were used to prepare the stock solutions of silver nanoparticles. Liquid chromatography‒mass spectrometry revealed the presence of substances, such as eleutheroside A and eleutheroside E, in the silver nanoparticles. These green-synthesized silver nanoparticles exhibited enhanced antibacterial activity against various bacteria [169].

Furthermore, nanoparticles with unique properties, including magnetic nanoparticles and electrospun nanofibers, have been utilized for nanodelivery. For example, Fe_3_O_4_ magnetic nanoparticles exhibit superparamagnetism that enables precise control by external magnetic fields and achieves targeted drug delivery to cancer cells. Moreover, under an alternating magnetic field, these magnetic nanoparticles can generate localized heat, holding potential applications, such as hyperthermia treatment in tumor tissues. For example, Khan et al. developed a formulation of curcumin prepared using superparamagnetic iron oxide nanoparticles as a nontoxic biologically active anti-inflammatory/anticancer agent. This formulation successfully improved the delivery efficiency of curcumin, especially for pancreatic tumors [170]. In another study conducted by Kaiping Wang, the anticancer drug paclitaxel and Fe_3_O_4_ magnetic material were co-encapsulated within the polylactic acid-glycolic acid copolymer nanoparticles. These coloaded drug nanoparticles induced apoptosis of A549 cells by disrupting lysosomes and activating the mitochondrial pathways, demonstrating a high anticancer activity [171]. Electrospinning technology is a method for preparing porous and spatially interconnected nanofibers. During this process, electrospinning involves the ejection and spinning of a charged polymer solution, forming nanofibers with diameters in the nanoscale range on a receiving device. The resulting nanofibers exhibit high porosity, large surface area, uniformity, and ability to mimic the extracellular matrix, making them suitable in tissue engineering, wound dressings, drug carriers, and other biopharmaceutical fields. This technology can be used for the production of nanofibers using effective traditional Chinese herbal materials, offering potential for the development of new wound healing materials and further leveraging the role of TCM in the promotion of wound healing and tissue repair. One study designed a “sandwich-like” structured electrospun nanofiber wound dressing for diabetes. It involved a hydrophilic polyvinyl alcohol inner layer loaded with metformin hydrochloride to facilitate diabetic wound healing. The middle layer, a composite membrane dressing, demonstrated excellent mechanical properties resembling human skin’s tensile performance. The hydrophobic polylactic acid outer layer contained erythromycin and puerarin, which demonstrated significant antibacterial properties [172]. These features make the diabetes wound dressing promising for medical applications.

### Nanoparticles associated with herbal small molecule compounds

Herbal small molecule compounds play a vital role in traditional medicine and are widely employed globally, including glycosides [173], flavonoids [174], Terpenoids [175], phenylpropanoids [176], alkaloids [177], and phenols [178]. Furthermore, these herbal extracts are considered essential sources for new drug development because of their diverse compounds with unique structures and considerable biological activities [17, 179]. Unfortunately, several purified compounds from these herbal extracts face limited absorption upon oral administration, imposing severe restrictions on their clinical applications [180, 181]. Through a bibliometric analysis, some notable Chinese herbal compounds were identified in the field of nanoparticles, including curcumin, berberine, resveratrol, quercetin, celastrol and others.

Curcumin, a compound derived from the Chinese herb turmeric, possesses a range of biological effects, including anti-inflammatory, anti-oxidant, and anti-cancer activities. However, curcumin has limited water solubility and chemical instability, leading to restricted bioavailability. Nanoparticle preparation represents an effective strategy for enhancing the solubility and bioavailability of curcumin. Strategies based on turmeric nanovesicles use nanovesicles extracted from fresh turmeric, with mass spectrometry identifying curcumin as a major component. The oral administration of turmeric nanovesicles exerts anti-inflammatory effects via mechanisms including the repair of damaged intestinal barriers, modulation of gut microbiota, and regulation of macrophage phenotypes [71]. Furthermore, strategies involving liposome and chitosan encapsulation have been utilized in curcumin delivery, employing various preparation methods and therapeutic applications. For example, a liposome nanoparticle, constructed by fusing the red blood cell membrane with a phospholipid bilayer, encapsulates curcumin in the hydrophobic region of the bilayer for delivery into macrophages to modulate macrophage polarization. This approach effectively alleviates excessive inflammation in diabetic wound healing and considerably promotes diabetic wound closure [182]. Furthermore, a layered nanoparticle system, comprising curcumin, zein, hyaluronic acid, piperine, and chitosan, has been reported. Through a layer-by-layer encapsulation approach, this system successfully delays the release of curcumin and piperine [183]. Moreover, gold and silver nanoparticles are commonly used to load curcumin. For example, curcumin-coated gold nanoparticles, through a green synthesis approach, can induce apoptosis in human renal cancer A498 cells [184]. Curcumin silver nanoparticles have been successfully encapsulated in electrospun nanofiber membranes, exhibiting biocompatibility and broad-spectrum antibacterial activity [79]. Water solubility and excellent antioxidant properties have been achieved through the combination of curcumin and iron in the preparation of iron–curcumin nanoparticles (Fe–Cur NPs). These particles effectively protect cells from oxidative stress damage. Similarly, Fe–Cur NPs can alleviate acute lung injury by clearing intracellular ROS and coreducing inflammation [75]. Another study reported the development of targeted biomimetic nanoparticles. These nanoparticles comprised curcumin-modified Cu2-xSe nanoparticles encapsulated in the macrophage membrane and enabled targeted drug delivery to mitochondria, improving neuronal mitochondrial function and thus enhancing the treatment of Parkinson's disease [76].

Berberine, an alkaloid with a rich history of use in TCM and Ayurvedic medicine, is known for its applications in clearing heat, detoxification, and antibacterial therapies. However, its limited water solubility results in low bioavailability when administered orally. Furthermore, it may exhibit toxicity at high doses, thereby restricting its clinical benefits. To overcome these challenges, researchers have developed various nanotechnology-based strategies for berberine. For instance, Dua et al. developed a novel drug delivery system known as berberine phytantriol liquid crystal nanoparticles (BP-LCNs), which encapsulated berberine within soluble, biodegradable polymers to improve its bioavailability, enhance the therapeutic efficacy, and reduce drug toxicity [80]. Berberine is an alkaloid compound that can react with molecules such as saponins and organic acids to produce amphiphilic substances, which can self-assemble into nanoparticles, and promote the oral absorption of the active ingredients. Inspired by the combination of traditional Chinese herbs such as berberine and citrus aurantium, Professor Le Hai Min and his team at the Beijing University of Chinese Medicine have developed a series of self-assembly strategies based on berberine. Berberine and cinnamic acid self-assemble directly into ordered cinnamic acid-berberine nanoparticles (CA-BBR NPs) via noncovalent bonds such, as electrostatic interactions, π–π stacking, and hydrophobic interactions. These CA-BBR NPs demonstrate excellent biocompatibility and sustained release properties, effectively adhering to and penetrating bacteria to combat multidrug-resistant *S. aureus* [91]. Furthermore, carboxyl groups in berberine can react with berberine under acid‒base neutralization reactions. Based on this finding, carrier-free binary nanoparticles of gallic acid-berberine (GA-BBR NPs) were prepared using an efficient “one-pot” method. These GA-BBR NPs self-assemble through electrostatic interactions, π–π stacking, and hydrophobic interactions [87]. Another study revealed that two natural plant compounds, berberine and hesperetin, directly self-assembled into carrier-free dual-function spherical berberine-hesperetin nanoparticles (BBR-HST NPs) through noncovalent bonds such as electrostatic interactions, π–π stacking, and hydrogen bonds. BBR-HST NPs exhibited synergistic anti-inflammatory effects, effectively modulating the immune environment and repairing damaged intestinal barriers, exhibiting significant advantages in the treatment of ulcerative colitis and inflammation as compared to equivalent doses of BBR and HST [88]. Furthermore, two natural compounds with antimicrobial and anti-inflammatory properties, berberine and sanguinarine, could self-assemble directly into carrier-free binary small molecule berberine-sanguinarine (BA-SAN) hydrogels through noncovalent bonds, such as electrostatic attraction, π–π stacking, and hydrogen bonding. Moreover, the BA-SAN hydrogel is suitable for wound dressings, inhibiting bacteria, reducing wound inflammation, and promoting wound healing, and it demonstrated good biocompatibility [89]. The integration of self-assembly strategies with nanodelivery systems has led to the development of more potent drug delivery systems. Jing Zhao et al. isolated and purified natural nanoparticles from astragalus extracts. These nanoparticles were self-assembled from proteins in coptis extracts and served as a natural nanodrug delivery system, significantly improving the pharmacokinetics of oral berberine [81]. Researchers have also developed various targeted delivery systems for transporting berberine. Photodynamic therapy has emerged as a novel cancer treatment method. Researchers developed a tumor-targeted drug by combining berberine, as an oxygen and immune microenvironment modulator, with the near-infrared photodynamic dye IR68 that was chemically modified with DSPE-PEG2000 liposomes. Berberine in liposomes reversed tumor hypoxia and enhanced reactive oxygen species generation and T-cell infiltration, thereby reducing the resistance to photodynamic immunotherapy [82]. Furthermore, exosomes were extracted from M2 macrophages and used as carriers to effectively accumulate berberine at the spinal cord injury sites. This exosome-based delivery system, using M2 macrophage exosomes as carriers, can serve as an anti-inflammatory drug delivery system for treating secondary spinal cord injuries [83].

Quercetin, a flavonol compound, is widely distributed in various plant tissues, such as the stems, bark, flowers, leaves, buds, seeds, and fruits, primarily in the form of glycosides. This compound exhibits diverse biological activities, including anti-inflammatory, anti-bacterial, anti-viral, and anti-cancer properties, as well as potential applications in the prevention and treatment of cardiovascular and cerebrovascular diseases. Nevertheless, its limited solubility and bioavailability have constrained its clinical utility. The preparation of quercetin–chitosan derivative-based nanoparticles through electrostatic interactions enhances the stability and bioavailability of quercetin, thereby improving its efficacy in treating intestinal mucosal inflammation [105]. In another study, quercetin was successfully loaded into extracellular vesicles derived from human mesenchymal stem cells using electroporation. These vesicles are characterized by high production capacity and low immunogenicity, making them an ideal carrier for certain type 2 diabetes therapeutic agents. This novel research indicates that quercetin-loaded extracellular vesicles can more effectively suppress β-cell apoptosis and promote insulin secretion, thereby alleviating type 2 diabetes [76, 103]. Moreover, it quercetin-modified ultrasmall Cu2-xSe nanoparticles can activate the Nrf2/Keap1/p62 signaling pathway in neurons, thereby improving the motor dysfunction in patients with Parkinson’s disease [76]. In another study, quercetin was associated with the antioxidant cerium dioxide nanoparticles for the design of a novel nanodrug with bidirectional regulation of macrophage differentiation. This design reduced the M1/M2 ratio in macrophages, inducing M1-to-M2 differentiation and thereby mitigating inflammation, offering a new periodontitis treatment strategy [104]. Moreover, recent research by Zhu Chunling introduced a unique self-assembled nanoparticle composed of the small peptide amino acid molecules 9-fluorenylmethyloxycarbonyl-tryptophan (Fmoc-Trp), Fe^2+^, and quercetin (Que). The assembly relied on coordination, electrostatic attraction, and hydrophobic interactions between these components, resulting in hollow-structured Fmoc-Trp-Fe-Que nanoparticles. This innovative assembly method transformed quercetin from a mere drug payload into a fundamental building unit for nanodrugs. This approach achieved efficient quercetin loading, enhancing its water solubility, resistance to degradation, antioxidant capacity, bioavailability, blood‒brain barrier penetration, and anti-Alzheimer’s disease performance [102].

Celastrol, a natural compound derived from *Tripterygium wilfordii*, has been extensively studied for its therapeutic potential in various diseases. However, its clinical utility has been hampered by its inherent toxicity to humans. The primary active constituents of *T. wilfordii* include triptolide and celastrol, both of which possess multifaceted pharmacological properties, including anti -rheumatic, anti-cancer, and proteinuria-reducing effects. To overcome the safety concerns associated with celastrol, numerous studies have explored the use of nanotechnology for drug delivery. For instance, Ruixing Liu designed a nanoformulation loaded with celastrol to circumvent ocular drug delivery barriers, reduce dosing frequency, and mitigate drug toxicity. This approach effectively suppressed corneal stromal fibrosis [92]. Another study successfully encapsulated celastrol into PEG-PCL nanoparticles, and these drug-loaded nanomicelles ameliorated inflammation and metabolic disruptions in obese mice, preventing potential gastrointestinal irritation and damage associated with celastrol monotherapy [93]. Several research investigations have demonstrated the advantages of targeted nanodelivery systems to minimize celastrol distribution in organs, thus mitigating multiorgan toxicity. One study developed a ROS-responsive polymer-based celastrol drug delivery micelle, which selectively accumulated and released celastrol at the site of inflammation in collagen-induced arthritis mice, effectively inhibiting rheumatoid arthritis progression [94]. Ling Guo et al. constructed a mesangial cell-targeted drug delivery system using albumin nanoparticles. They reported that celastrol-loaded albumin nanoparticles penetrated the fenestrated endothelium and accumulated in the mesangial cells, significantly enhancing the anti-inflammatory, anti-proliferative, and anti-mesangial matrix deposition effects of celastrol. This approach improved the in vivo distribution of celastrol while reducing the associated side effects [95]. Moreover, enzyme-responsive nanoparticles were employed to selectively induce apoptosis of osteoclasts and macrophages in the joints of rheumatoid arthritis by delivering celastrol [95]. Furthermore, another study also reported that folic acid-modified ROS-responsive celastrol nanoparticles were designed to alleviate rheumatoid arthritis symptoms [96]. Moreover, injected celastrol nanoparticles have maintained sustained drug concentrations in melanomas, inducing strong immunogenic cell death and programmed death-ligand 1 downregulation, effectively inhibiting tumor growth [98].

Similar to celastrol, triptolide, another bioactive compound derived from the Thunder God Vine, has been explored using a range of drug delivery strategies, including lipid-based systems, extracellular vesicles, and self-assembly methods. Triptolide/Ce6 liposome nanoparticles have been utilized to enhance the penetration and retention effect for triptolide accumulation at tumor sites. Upon laser irradiation, the photosensitizer Ce6 generates reactive oxygen species, further oxidizing unsaturated phospholipids, leading to liposomal disruption and triptolide release for efficient hepatocellular carcinoma therapy [114]. Extracellular vesicles, specifically dendritic cell-derived exosomes, have been explored as nanocarriers for triptolide. These dendritic cell-derived exosomes have a natural affinity for their parent cells, thereby enabling targeted drug delivery of triptolide and avoiding mononuclear phagocyte system uptake and immunological clearance. This approach has shown successful and safe therapeutic outcomes in mouse models of autoimmune diseases [113]. Furthermore, triptolide has been encapsulated within tumor necrosis factor-related apoptosis-inducing ligand (TRAIL)-engineered exosomes by using ultrasound and ultraspeed centrifugation techniques. TRAIL can selectively bind to death receptor 5, which is highly expressed in human melanoma cells, leading to targeted apoptosis, whereas triptolide release triggers the apoptosis signaling pathway [185]. One study reported the development of a self-assembled glutathione-activated triptolide nanoparticle (CyssTPN), which is carrier-free. In comparison to carrier-based triptolide delivery systems, CyssTPN significantly reduced toxicity and achieved increased drug release under glutathione stimulation in tumor tissues, thereby enhancing the therapeutic efficacy [106]. In summary, these nanotechnological advancements hold great promise for enhancing the therapeutic efficacy of celastrol and triptolide while simultaneously mitigating their toxicity. These studies provide strong support for the clinical application of these natural compounds in the treatment of various diseases.

Currently, research on plant nanoparticles has progressed to a certain extent, although investigations in the field of medical diseases remain at the stage of cell culture or animal experiments. Despite the majority of experimental results demonstrating the biocompatibility of plant nanoparticles, few studies have reported adverse reactions to TCM nanoparticles, primarily attributed to the nanoformulation of herbal medicines leading to the accumulation of drug concentration [21], coupled with the inherent toxicity of certain nanocarrier materials. Owing to the heterogeneity of the human body and experimental models, the likelihood of translating herbal nanoparticles into human clinical trials is low, hindering the establishment of effective and safe assessments in the human body. Furthermore, the complex manufacturing process of nanotechnology makes it large-scale clinical implementation challenging, resulting in uncertainties and challenges between laboratory experiments and clinical applications. Nevertheless, there remains optimism for clinical research on plant nanoparticles, particularly with breakthroughs in certain technological methods, such as CDs and self-assembly techniques, which simplify nanoparticle fabrication and reduce reliance on traditional gold or silver nanoparticle delivery systems, thereby minimizing the development of adverse effects on the human body. Moreover, the use of some plant nanoparticles in tumor diseases provides insights for other diseases; for example, albumin-bound paclitaxel, a nanoformulation of paclitaxel bound to albumin, has shown promising results in improving survival rates in pancreatic cancer clinical trials [186]. Furthermore, given the safety of topical applications of plant nanomedicines, our research has identified their potential in wound healing and treatment of psoriasis, osteoarthritis, and other conditions [61, 77, 87]. Therefore, the development of novel plant nanoparticle dressings for surface-related diseases holds great promise for breakthroughs in clinical research.

To our knowledge, plant nanomedicine is a highly anticipated frontier in the interdisciplinary field of medicine and engineering. Many medicinal compounds in plants, such as curcumin and berberine, have poor water solubility and low bioavailability [180, 181]. Therefore, medical or pharmacological researchers must not only consider the effects of a single plant component on disease models, but also address the key areas, such as compound stability, distribution and pharmacokinetics. Breakthroughs can be achieved by medical or pharmacological researchers via interdisciplinary research. In China, some clinically used medicinal plants in their fresh form or after boiling in various decoctions induce significant responses in the human body. However, when a single major component of these herbs is extracted and purified, significant effects are often not seen. Could this be because of the nanovesicular form of the fresh herbal medicine or to the formation of nanoparticles after boiling, which aid drug delivery? Therefore, integrating nanotechnology into pharmacological research may lead to more satisfactory results. Our research highlights the hotspots in plant nanomedicine research, including countries, institutions, and experimental teams, providing guidance for researchers seeking collaborations.

## Limitation

Contrary to traditional review articles, our study provides a comprehensive and quantitative analysis of research focuses, patterns, and collaborative efforts in the field of herbal nanoparticles. This approach enables a better understanding of the development in this field. However, our research has several limitations that need to be acknowledged. First, because of software limitations, we focused solely on publications from the WOSCC database, excluding nonEnglish publications, which may introduce publication bias. Second, citation data are subject to temporal influence, with older papers frequently receiving more citations than newer papers. Therefore, some outstanding papers might not receive their due recognition because of their relatively shorter availability. Furthermore, the common practice of citing review articles rather than original research may introduce bias. Moreover, using different combinations of literature analysis software may lead to omission of information, causing slight variations in the results. In the present study, we categorized the discussion of research hotspots that might possibly limit our discussion to some extent and not entirely represent the full landscape of herbal nanoparticles. Despite these limitations, our research provides a comprehensive survey of hotspots and trends in the field of herbal nanoparticles, offering valuable insights for current and future research work.

## Conclusion

This pioneering study has provided an indepth analysis of research directions and future prospects in the field of herbal nanoparticles. Overall, the number of publications in this research area between 2004 and 2023 has rapidly increased. China has exhibited significant productivity in this specific research field, emerging as the most influential nation. Meanwhile, countries using traditional herbal medicine, such as India, Iran, and Arabic nations, have also become popular publishing nations. Despite having lower publication numbers, the United States engages in extensive collaborations with these countries. Journal analysis highlights influential and highly productive journals in the field, with a particular focus on the area of plant nanomedicine, driving its advancement. The results of the keyword clustering analysis reveal that research in this field primarily centers on green synthesis, curcumin, wound healing, drug delivery, and CDs, prominently featuring new synthesis methods, drug delivery, herbal small molecule compounds, and applications of herbal nanoparticles enabled by nanotechnology. These timely findings provide fresh insights for the development of TCM, helping researchers to select appropriate journals for publication, identify potential collaborators, and understand hot issues and cutting-edge areas, thus propelling the advancement of this field.

### Supplementary Information

**Additional file 1: DAT.** Data extracted from Web of Science.

**Additional file 2: Table S1**. Keywords clusters information by Vosviewer.

## Data Availability

The dataset supporting the conclusions of this article is included within the article (Additional file 1).

## Abbreviations

WoSCC: Web of Science Core Collection
PDT: Photodynamic therapy
PTT: Photothermal therapy
PDVLNs: Plant-derived vesicle-like nanoparticles
LEVNs: Lemon-derived extracellular vesicles
CDs: Carbon dots
TCM: Traditional Chinese medicines
Fe-Cur NPs: Fe-curcumin nanoparticles
BP-LCNs: Berberine Phytantriol Liquid Crystal Nanoparticles
GA-BBR NPs: Cinnamic acid-berberine nanoparticles
GA-BBR NPs: Gallic acid-berberine nanoparticles
BBR-HST NPs: Berberine-hesperetin nanoparticles
BA-SAN: Berberine-sanguinarine
Fmoc-Trp: 9-Fluorenylmethyloxycarbonyl-tryptophan
Que: Quercetin
PD-L1: Programmed death-ligand 1
TRAIL: Tumor necrosis factor-related apoptosis-inducing ligand
CyssTPN: Glutathione-activated triptolide nanoparticle
