# Supplementary figures and images for: Collision of herbal medicine and nanotechnology: a bibliometric analysis of herbal nanoparticles from 2004 to 2023
Source: J Nanobiotechnology. 2024 Apr 1;22:140. doi: 10.1186/s12951-024-02426-3 (PMC10983666; doi:10.1186/s12951-024-02426-3)

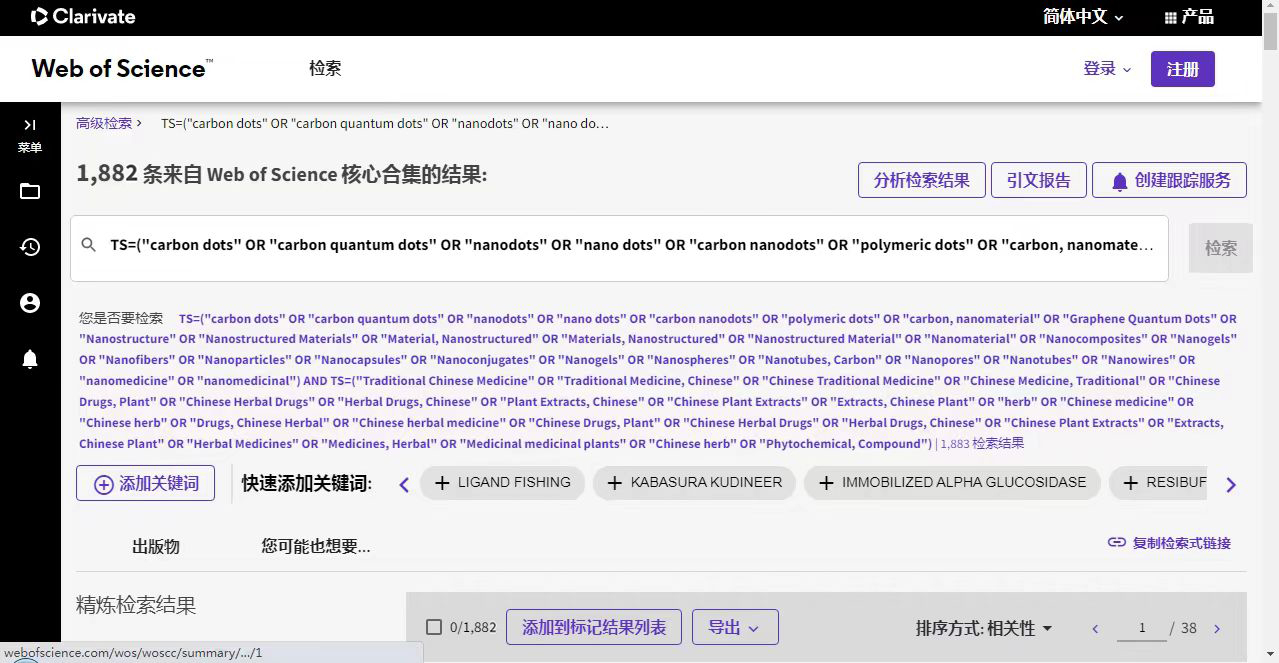

Supplement: Supplementary file 1 — Additional file 1: DAT. Data extracted from Web of Science. [file 12951_2024_2426_MOESM1_ESM.zip › Supplementary files/微信图片_20231114022142.jpg]
