# Supplementary material for: Collision of herbal medicine and nanotechnology: a bibliometric analysis of herbal nanoparticles from 2004 to 2023
Source: J Nanobiotechnology. 2024 Apr 1;22:140. doi: 10.1186/s12951-024-02426-3 (PMC10983666; doi:10.1186/s12951-024-02426-3)
Supplement: Supplementary file 2 — Additional file 2: Table S1. Keywords clusters information by Vosviewer. [file 12951_2024_2426_MOESM2_ESM.docx]

Table S1. Keywords clusters information by Vosviewer

| cluster 1 | traditional chinese medicine; quercetin; flavonoids; capillary electrophoresis; electrochemical sensor; nanomaterials; reactive oxygen species; baicalin; graphene; graphene oxide; inhibitor screening; voltammetry; carbon nanotube; chinese herbal medicines; magnetic solid-phase extraction; mass spectrometry; nanoformulations; surface-enhanced raman spectroscopy; alpha-glucosidase; amperometric detection; chemiluminescence; copper; fluorescence; honokiol; luteolin; mycotoxins; pesticide residues; phytochemical analysis; aptasensor; baicalein; carbon nanotubes; dispersive solid-phase extraction; drug screening; electrochemistry; lead; magnolol; modified electrode; multi-walled carbon nanotubes; nanocomposite; pesticides; rutin; sers; |
| --- | --- |
| cluster 2 | green synthesis; silver nanoparticles; gold nanoparticles; antibacterial; antibacterial activity; magnetic nanoparticles; antioxidant activity; biosynthesis; bioactive compounds; agnps; essential oil; ligand fishing; anticancer activity; pharmacology; characterization; photocatalysis; plant extract; rosmarinic acid; chitosan nanoparticles; obesity; silver nanoparticle; andrographolide; molecular docking; zeta potential; aunps; drug discovery; gc-ms; gene expression; larvicidal activity; medicinal plant; secondary metabolites; synthesis; toxicology; transmission electron microscopy; zinc oxide nanoparticles; zno nanoparticles; |
| cluster 3 | apoptosis; berberine; breast cancer; celastrol; triptolide; anticancer; autophagy; blood-brain barrier; hepatocellular carcinoma; combination therapy; nanostructures; rheumatoid arthritis; angiogenesis; glioma; mechanism; self-assembly; antitumor; borneol; doxorubicin; resveratrol; ursolic acid; safety; controlled release; in vitro; in vivo; liposome; metastasis; nanosuspension; ros; selenium nanoparticles; tem; bufalin; folic acid; gastric cancer; targeted delivery; traditional medicine; |
| cluster 4 | nanoparticles; herbal medicine; nanoparticle; cancer; liposomes; cancer therapy; chinese medicine; drug delivery system; phytochemical; emodin; colorectal cancer; immunotherapy; chemotherapy; gambogic acid; inflammatory bowel disease; nanocarrier; drug resistance; micelles; nanoemulsions; photodynamic therapy; realgar; |
| cluster 5 | antioxidant; wound healing; phytochemicals; toxicity; antimicrobial; biocompatibility; hplc; polysaccharide; encapsulation; extraction; hemostasis; herb; stability; biological activity; biomedical applications; flavonoid; health; pharmacological activity; phytochemical compounds; polyphenol; synergistic effect; |
| cluster 6 | carbon dots; anti-inflammatory; herbal medicines; herbs; adsorption; polyphenols; biochar; traditional chinese medicines; bioimaging; ferulic acid; magnetic molecularly imprinted polymers; plant extracts; solid phase extraction; chinese herbal medicine; drugs; metal nanoparticles; chinese herbs; heavy metals; hexavalent chromium; |
| cluster 7 | drug delivery; curcumin; chitosan; natural products; inflammation; nanoemulsion; plga; psoriasis; anti-cancer; nanoformulation; mesoporous silica nanoparticles; nanocapsules; turmeric; herbal drug; melanoma; microencapsulation; tetrandrine; thymoquinone; |
| cluster 8 | medicinal plants; antioxidants; covid-19; medicinal herbs; polysaccharides; novel method; sars-cov-2; immunity; clinical trials; eco-friendly; herbal; innovative technology; kabasura kudineer; sem; shikonin; silica; silver; |
| cluster 9 | nanotechnology; bioavailability; pharmacokinetics; oxidative stress; alzheimer's disease; drug delivery systems; nanocarriers; biological activities; herbal drugs; puerarin; solubility; traditional chinese medicine (tcm); oral bioavailability; active ingredients; dissolution; nanocrystals; parkinson's disease; |
| cluster 10 | cytotoxicity; electrospinning; wound dressing; solid lipid nanoparticles; nanofiber; sustained release; tissue engineering; bone tissue engineering; icariin; antitumor activity; lung cancer; silk fibroin; solid lipid nanoparticle; |
| cluster 11 | nanomedicine; antimicrobial activity; diabetes; biomaterials; |
| cluster 12 | liver cancer |
